# Dwarf Kiwi (*Actinidia arguta* Miq.), a Source of Antioxidants for a Healthy and Sustainable Diet

**DOI:** 10.3390/molecules27175495

**Published:** 2022-08-26

**Authors:** Patricia Garcia-Herrera, Helayne A. Maieves, Erika N. Vega, María Luisa Perez-Rodriguez, Virginia Fernandez-Ruiz, Amaia Iriondo-DeHond, Maria Dolores del Castillo, Maria Cortes Sanchez-Mata

**Affiliations:** 1 Departament of Nutrition and Food Science, Faculty of Pharmacy, University Complutense of Madrid, Pza, Ramón y Cajal s/n, 28040 Madrid, Spain; 2 Departament of Nutrition, Federal University of Pelotas, Rua Gomes Carneiro n.1, Pelotas 88630, Brazil; 3 Institute of Food Science Research (CIAL, UAM-CSIC), Nicolas Cabrera, 9, 28049 Madrid, Spain

**Keywords:** *Actinida arguta*, antioxidants, dwarf kiwi, baby kiwi, kiwi berry, intracellular ROS, phenolic compounds, QUENCHER

## Abstract

The feasibility of using dwarf kiwi fruits (*Actinia arguta* Miq.) as a healthy and sustainable food, compared to other types of commercial kiwi fruits, was evaluated in the present study. The overall antioxidant capacity of these fruits was assessed by either extraction-dependent methods (ABTS, ORAC) or the direct method called Quick, Easy, New, CHEap, Reproducible (QUENCHER) (DPPH, FRAP, Folin–Ciocalteu), applied for the first time to analyze kiwi fruits. With this methodology, all the molecules with antioxidant capacity are measured together in a single step, even those with high molecular weight or poor solubility in aqueous extraction systems, such as antioxidant dietary fiber. The effect of kiwi extracts on physiological and induced intracellular reactive oxygen species (ROS) production on IEC-6 cells was also analyzed, as well as total phenolic content (TPC) by Fast Blue BB, flavonols, hydroxycinnamic acids, and hydroxybenzoic acids. *A. arguta* fruits showed the highest values in all the antioxidant assays, being remarkably higher than the other kiwi species for Q-FRAP and Q-DPPH. Dwarf kiwi showed the highest potential in reducing physiological ROS and the highest values of TPC (54.57 mgGAE/g), being hydroxybenzoic acids the main phenolic family found (2.40 mgGAE/g). Therefore, dwarf kiwi fruits are a natural source of antioxidants compared to conventional kiwi fruits, being a sustainable and healthy alternative to diversify fruits in the diet.

## 1. Introduction

The genus Actinidia has 76 species, but only two of them, *Actinidia deliciosa* and *Actinidia chinensis* (golden kiwi fruit), are produced on a commercial scale. Other species of the genus include *Actinidia arguta* (dwarf or baby kiwi fruit), *Actinidia purpurea* (purple kiwi fruit), *Actinidia kolomikta* (arctic kiwi fruit), *Actinidia eriantha* (velvet vine), *Actinidia polygama* (silver kiwi fruit), and *Actinidia melanandra* (red kiwi) [1]. Many of these fruits have great nutritional potential due to their high content of vitamins, such as vitamin C, E, K, and B9, in addition to dietary fiber, potassium, and other minerals, as well as various phytochemicals such as carotenoids (lutein and *β*-carotene) and phenolics such as flavonoids, including anthocyanins [2]. Many of these bioactive molecules have shown interesting health-promoting properties such as being antioxidants for the human body by different mechanisms, and are responsible for some biological properties attributed to kiwi fruits in the protection of the body against chronic diseases, especially those related to oxidative equilibrium in tissues [3].

Consumption of exotic fruits which are not commercially grown is a good choice to diversify the diet and combat malnutrition, which includes both under-nutrition and obesity. The two forms of malnutrition can be present in the same community [4,5]. The “Farm to Fork” strategy [6] raises the need for a sustainable food system that guarantees food security and nutrition for all people at the present time without compromising future generations. “Sustainable Healthy Diet” is a concept among the objectives proposed for a sustainable food system. The main aim of this system is to prevent malnutrition, decrease the risk of chronic diseases related to an unhealthy diet, as well as preserve the biodiversity and health of the planet [7]. Balanced and healthy diets can help reduce food overconsumption, which is currently responsible of a huge amount of food waste [4]. The Global Burden of Disease Study (2019) [8] reports that a low intake of vegetables is related to higher mortality worldwide. The World Health Organization (WHO) recommends increasing the consumption of fruits and vegetables (at least 400 g or five portions of fruits and vegetables a day), including vegetables at all meals (a varied selection of them) [9]. Plant-based foods produce the lowest greenhouse gas emissions, require low land use and energy, and have a lower impact on soil acidification and eutrophication than foods from animal origin [10]. In addition, the consumption of unprocessed or minimally processed foods is promoted, so in this sense, fresh vegetables are the main components of a healthy and sustainable diet.

In this context, dwarf fruits are potential candidates to be included in a healthy and sustainable diet. The most commercialized species is *Actinidia arguta* Miq. This fruit can present different designations: “dwarf kiwis”, “kiwi berry”, “mini kiwi”, “baby kiwi”, or “kiwiño”; they weigh between 6 and 14 g and can have different shapes. The pulp is sweet and very similar to conventional kiwi, and they can have a color hue that varies between green, yellow, and red. An important difference of the dwarf kiwi in relation to the common fruit is its green skin; since it does not present brownish fuzz, it can be consumed entirely. From an agricultural point of view, this type of fruit is more resistant to low temperatures. In the past few years, it has been cultivated mainly in New Zealand, the United States, Japan, Chile, and some European countries, such as France and Portugal [11,12]. Their different aspects from the traditional kiwi fruit, particularly the fact that they can be eaten without peeling and in a single bite, make them very attractive, especially for children. Therefore, the consumption of this type of kiwi is an important strategy for the improvement of human health status [13]. 

The measurement of antioxidant capacity in plant materials is mostly limited to extraction-based procedures that have several limitations. Depending on the polarity of each independent compound, they can be soluble or insoluble in a given solvent. Furthermore, antioxidants in foods may be generally found as free and bound forms (including chemically bound to high-molecular-weight macromolecules such as dietary fiber, ionically bound to the food matrix, physically entrapped in the food matrix, or physically entrapped in various cellular structures) [14]. With all these possibilities, it is difficult to obtain a high efficiency of extraction using only one solvent or a mixture of solvents because plant material presents lipophilic or hydrophilic molecules, which could be bound to the antioxidant components. Thus, antioxidant capacity is frequently underestimated [15]. This is the case of, for example, the so-called antioxidant fiber; this concept is related to the consideration of dietary fiber and antioxidants together in terms of health studies. According to Saura-Calixto (2011) [16], around 50% of total dietary antioxidants, mainly phenolics, traverse the small intestine linked to dietary fiber. These author reports proanthocyanidins, hydrolyzable phenols, ferulic acid, and o-hydroxybenzoic acid as some of the main phenolics associated with fiber in fruits. The QUENCHER procedure (QUIck, Easy, New, CHEap, and Reproducible), reported for the first time by Serpen et al. (2007) [17] and optimized by Del Pino et al. (2015) [18] has been proposed to evaluate the antioxidant capacity without any extraction. Using this approach, it has been shown that as the solid food (reduced to a very small particle size) is in direct contact with the radical reagent solution, soluble molecules exert their antioxidant capacity by quenching radicals present in the solvent according to the usual liquid–liquid reaction; at the same time, the insoluble part exerts its antioxidant capacity by means of the surface reaction occurring at the solid–liquid interface. In this way, the ability of QUENCHER methodology to evaluate the antioxidant capacity of compounds when they are still bound to the insoluble matrices, such as polymers, has been previously reported, assessing its suitability to globally evaluate the antioxidant capacity corresponding to extractable and non-extractable antioxidants [19].

The antioxidant capacity of kiwi fruit has been reported in several studies [20,21]; nevertheless, there is scarce literature comparing *A. arguta* Miq. to other species [22,23,24]. Thus, the objective of this work was to assess the antioxidant capacity of kiwi fruits by either extraction-dependent methods or novel QUENCHER direct methods in order to include the contribution of low-molecular-weight substances (such as phenolics), as well as poorly soluble or bound antioxidants. With this aim, the antioxidant properties of dwarf kiwi were compared to other species and varieties of commercial kiwi samples (green fruits of *A. deliciosa*, AD; green fruits of *A. deliciosa* cv. Hayward, HW; green fruits of *A. deliciosa* cv. Hayward, organic production, ECO; gold fruits of *A chinensis*, G) using both chemical in vitro assays and experiments on healthy intestinal cell lines.

## 2. Results and Discussion

### 2.1. Antioxidant Properties

Table 1 shows the antioxidant properties of samples measured by both extraction-dependent (ORAC, ABTS) and direct (Folin–Ciocalteu, DPPH and FRAP, through QUENCHER approach) methods. Higher values of antioxidant capacity were obtained by the QUENCHER methodology probably for two reasons: (1) the measurement of insoluble material and (2) the less selective extraction of components, including some hydrophobic antioxidants, especially in Q-DPPH and Q-FRAP assays, in agreement with previous studies [14]. Moreover, Q-Folin–Ciocalteu, Q-DPPH, and Q-FRAP methods are based on electron transfer mechanisms, while ABTS is based on hydrogen atom transfer and the ORAC method is based on radical peroxyl generation. The higher antioxidant capacity observed for the QUENCHER methodology could be related to a more complete reaction with antioxidant compounds, and also to the presence of antioxidant compounds acting by electron transfer mechanisms. *A. arguta* (AA) presented the highest antioxidant capacity measured by all the methods carried out, with significant differences (*p* < 0.05) between dwarf kiwi (AA) and the other species.

Zhang et al. (2021) reported the antioxidant capacity of eight Chinese baby kiwi varieties by peroxyl radical scavenging capacity assay, obtaining high values ranging from 1433.54 to 5143.67 µmol vitamin C equivalents (VCE)/100 g of fresh weight (FW) [24]. These data are not comparable to the presented values because they use another methodology and different expression of results; however, high variability within different varieties of kiwi fruits is evidenced, in agreement with this study. Wang et al. (2018) studied different Chinese varieties of kiwi and concluded that the antioxidant capacity was ranked in the following order: *A. arguta* > *A. chinensis* (gold) > *A. deliciosa* > *A. deliciosa* cv. “Hayward” [23]. This trend is in accordance with the results obtained in the present study.

Fruits of AA showed the highest antioxidant capacity by the direct Q-FRAP method (26.24 mg TE/g). The highest difference between AA and the other varieties was evidenced in the Q-DPPH assay and Q-FRAP, where it was more than twofold higher. It should be considered that Q-FRAP allows the measurement of hydrophilic and hydrophobic antioxidant molecules acting by electron transfer mechanisms. On the contrary, the method that presented the smallest difference was extraction-dependent ABTS, but even so, AA’s value (1.73 µmol TE/mg) was still significantly the highest (*p* < 0.05). For *A. deliciosa* kiwi fruits (AD), in the case of ORAC and ABTS assays, the highest value was found in the AD sample. *A. chinensis* (G) presented the highest value for Q-FRAP and Q-DPPH assays, and *A. deliciosa* var. Hayward (HW) showed higher values for the Q-Folin–Ciocalteu (Q-FC) assay. The antioxidant capacity of kiwi fruits can be attributed mainly to phenolics and other compounds such as ascorbic acid or carotenoids, among others. Ascorbic acid is present in kiwi fruits at a level of about 80–160 mg/100 g, with high variability due to genetic and environmental factors or ripening status factors [2]. Carotenoids (β-carotene, lutein) have been found in levels of 0.2–2.5 mg/100 g in different varieties of *A. deliciosa*, *A. chinensis*, and *A. arguta* kiwi fruits, with non-significant variations related to flesh color due to the much higher presence of chlorophylls in green kiwi fruits compared to yellow ones [25,26].

**Figure 1:**
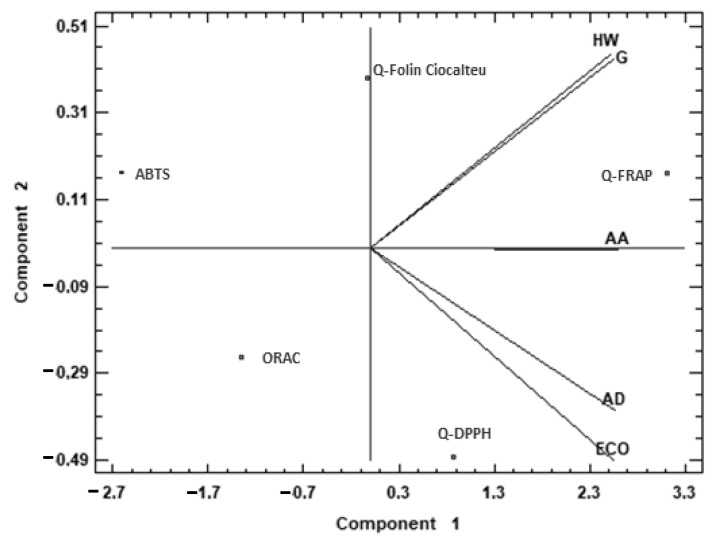
Principal component analysis (PCA) projection of two principal components on antioxidant capacity. Sample letters: AD: *A. deliciosa*; HW: *A. deliciosa* var. Hayward; ECO: *A. deliciosa* var Hayard eco; G: *A. chinensis*; AA: *A. arguta*.

To complete the comparison between the different species, a multivariate analysis was applied to the antioxidant capacity data (Figure 1). A principal component analysis (PCA) was performed, reducing the multi-dimensional structure of the data, which provided a two-dimensional map for explaining the observed variance.

Figure 1 shows the difference between the analyzed species using principal component analysis (PCA) based on the antioxidant capacity data. The two components of the PCA explained 97.72% of the total variance. Examination of the PCA graphs shows a similarity between HW and G, and between AD and ECO. The first principal component (95.17% of the total variance) was highly correlated with Q-FRAP, while it was negatively correlated with ABTS. The second principal component (2.55% of the total variance) was highly correlated with Q-FC and to a minor degree with Q-FRAP and ABTS, while it was negatively correlated with ORAC and Q-DPPH assays. HW and G were mostly characterized by the second component, while AD and ECO were negatively characterized by the second component. This means that AD and ECO were characterized by higher antioxidant capacity measured by Q-FRAP, and HW and G presented higher capacity measured by Q-FC. AA cannot be explained by the second component, but the first one explained its high antioxidant capacity measured by Q-FRAP.

In order to study these antioxidant properties on cellular models, prior to the evaluation of the effect of kiwi samples on the intracellular ROS formation, cell viability assays were performed to select non-cytotoxic concentrations of extracts. A concentration of 1 mg/mL (73% of cellular viability) was selected for the analysis of antioxidant capacity.

**Figure 2:**
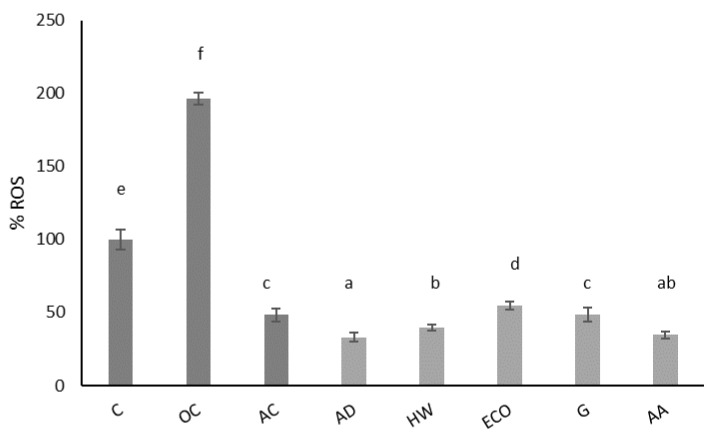
Effect of aqueous extracts obtained from different kiwi fruits (1 mg/mL) on the physiological ROS of IEC-6 intestinal cells. C: control—untreated cells; OC: oxidation control—tBOOH (1 mM); AC: antioxidant control—vitamin C (10 μg/mL); AD: *A. deliciosa*; HW: *A. deliciosa* var. Hayward; ECO: *A. deliciosa* var Hayard eco; G: *A. chinensis;* AA: *A. arguta*. Data are shown as the mean ± SD of three independent experiments. Different letters above columns indicate significant differences among treatments (Duncan test, *p* ≤ 0.05). Different shadow color has been used to differentiate controls and samples.

Figure 2 shows the effect of kiwi extracts on physiological ROS of IEC-6 cells after 24 h of incubation. This extract significantly reduced (*p* < 0.05) the physiological production of ROS compared to the control (untreated cells). A concentration of 1 mg/mL of kiwi gold (G) extract had a similar antioxidant effect (*p* > 0.05) as 10 μg/mL ascorbic acid, used as an antioxidant control. The best antioxidant effect was achieved with AD and AA, followed by HW, which did not present statistically significant differences (*p* < 0.05). These data confirm the antioxidant properties of the dwarf kiwi in intestinal cells and support its intake as an antioxidant fruit.

To the best of our knowledge, there are no studies regarding the effect of different kiwi samples on intracellular ROS of healthy intestinal cells. However, previous research has reported the effect of kiwi and other fruits and vegetables on intracellular ROS formation in hepatic cells (HepG2 cells). Kiwi fruits have shown a similar antioxidant capacity in HepG2 cells as honeydew, mango, lemon, orange, and cantaloupe [27]. The antioxidant properties were also evaluated in different kiwi berry samples on the physiological intracellular ROS of HepG2 cells. The results suggest that kiwi berry is a better source of antioxidants than common kiwi fruit at the cellular level [28].

**Figure 3:**
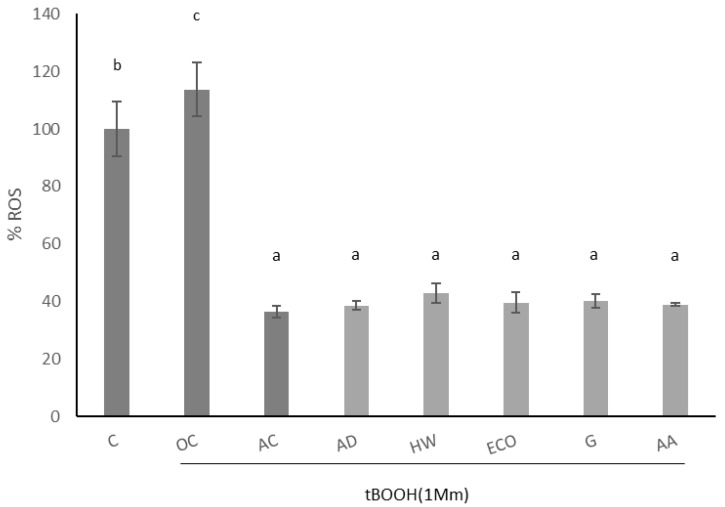
Effect of aqueous extracts obtained from different kiwi fruits (1 mg/mL) on induced ROS of IEC-6 intestinal cells. C: control—untreated cells; OC: oxidation control—tBOOH (1 mM); AC: antioxidant control—vitamin C (10 μg/mL); AD: *A. deliciosa*; HW: *A. deliciosa* var. Hayward; ECO: *A. deliciosa* var Hayard eco; G: *A. chinensis*; AA: *A. arguta*. Data are shown as the mean ± SD of three independent experiments. Different letters above columns indicate significant differences among treatments (Duncan test, *p* ≤ 0.05). Different shadow color has been used to differentiate controls and samples.

The effect of kiwi extracts on induced intracellular ROS of IEC-6 cells is shown in Figure 3. Oxidation was induced by 1 mM tBOOH, which significantly increased the production of intracellular ROS (*p* < 0.05). As expected, ascorbic acid significantly reduced the formation of induced ROS (*p* < 0.05). When intestinal cells were pre-treated with kiwi extracts (1 mg/mL), a significant reduction in induced ROS was observed (*p* < 0.05), similarly in all species.

An et al. (2016) evaluated the effect of various cultivars of kiwi berry on the induced ROS of RAW 264.7 mouse macrophages [29]. All three kiwi berry varieties studied (cv. Mansoo, cv. Chiak, and cv. Haeyeon) significantly reduced induced intracellular ROS in a dose-dependent manner. Kiwi berry extracts also showed anti-inflammatory properties in these cells by the inhibition of nitric oxide (NO) generation after induction with LPS. The authors explained that such cellular antioxidant effects may be attributed to their direct antioxidant capacity or to the inhibition of ROS generation via anti-inflammatory effects. In addition, the antioxidant capacity of kiwi juice has been evaluated in connective mouse tissue cells (L-929) under oxidation conditions induced by tBOOH [30]. Kiwi juice (1 mg/mL) showed lower antioxidant capacity than strawberry juice but higher antioxidant properties than peach juice.

Thus, kiwi extracts in this study showed in vitro antioxidant capacity and were effective against induced ROS in intestinal cells.

### 2.2. Phenolic Compounds

Table 2 shows the total polyphenolic content of kiwi samples as well as the studied phenolic families.

The Q-Fast Blue method was chosen for analysis of TPC to avoid interferences with Folin–Ciocalteu reagent, as indicated below. Contrary to what might be expected, Q-Fast Blue results were higher than Q-Folin–Ciocalteu results. This has also been described by other authors in fruits and juices [31], concluding that a ratio of Folin–Ciocalteu:Fast Blue may be attributed to the high presence of reducing non-phenolic compounds, while a ratio greater than 1 may be due to the higher presence of phenolic compounds. AA presented the highest value of Q-TPC, corresponding to 10.71 mg GAE/g (FW). This value is consistent with the antioxidant capacity shown previously. The TPC in this fruit according to Zuo et al. (2012) was 3.62 mg GAE/g (FW) [32]. Similar data were reported by other authors such as Wang et al. (2018) (3.23 mg/g FW) and Zhang et al. (2021), who reported that the TPC in this sample was 3.19 mg GAE/g (FW) [23,24]. Furthermore, AA has shown higher TPC values than other fruits, such as strawberries (10.71 vs. 1.5–2.5 mg GAE/g FW) [33].

Regarding phenolic families, hydroxybenzoic acids were higher than other families analyzed in all studied varieties. It would be expected that these compounds are important contributors to the antioxidant capacity. In the study carried out by Zhu et al. (2021) in conventional kiwi species marketed in Australia, gallic acid, protocatechuic acid, and 2,3-dihydroxybenzoic acid were present only in SunGold and Hayward samples, and the presence of these phenolic compounds may have contributed to their relatively higher antioxidant capacities [34]. Other studies reporting separate quantification of phenolic composition of kiwi berry have been recently published using UHPLC-PAD techniques [24,28]. In these studies, protocatechuic acid, caffeic acid, chlorogenic acid, and quinic acid were the predominant phenolics in kiwi berry, with values of 24.3–29.1 μg/g (fresh weight) as free phenols and 11.2–14.2 μg/g (fresh weight) as bound phenols. Other compounds identified and quantified in smaller amounts are the flavanols proanthocyanidin B2, proanthocyanidin C1, and (+)-gallocatechin, as well as the flavonols quercetin-3-*O*-galactoside, quercetin-3-*O*-rutinoside, and quercetin-3-*O*-glucoside. In the study of Zhang et al. (2021), flesh and peels of kiwi berry were compared, the concentration of phenolic acids being about three times higher in the peels than in the flesh [28]. From this, it can be stated that the intake of dwarf kiwi, as a whole fruit together with the peel, involves a nutritional advantage for a higher intake of phenolic compounds.

To complete the study of TPC and phenolic families of the different kiwis, a multivariate analysis (PCA) was performed, which provided a two-dimensional map for explaining the observed variance.

**Figure 4:**
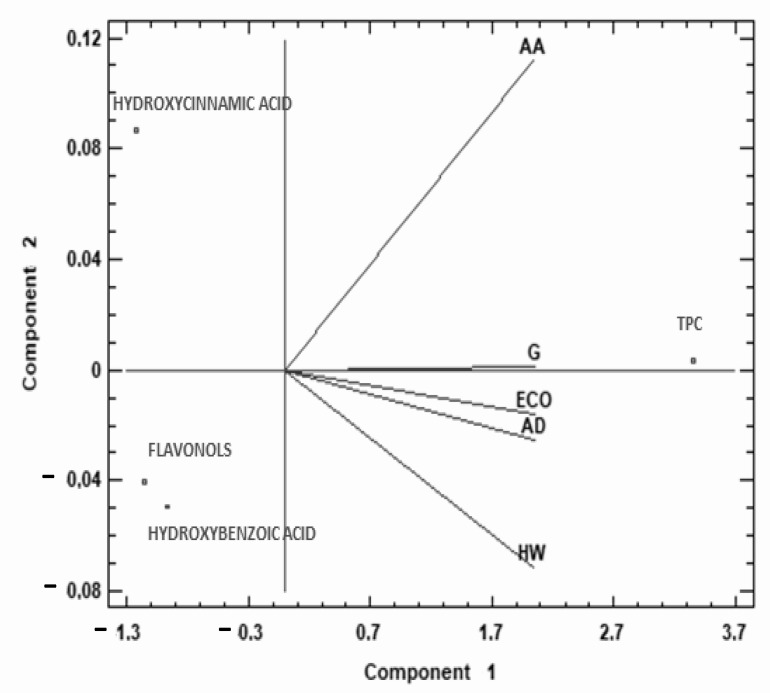
Principal component analysis (PCA) projection of three principal components on TPC and phenolic acids. Sample letters: AD: *A. deliciosa*; HW: *A. deliciosa* var. Hayward; ECO: *A. deliciosa* var Hayard eco; G: *A. chinensis*; AA: *A. arguta*.

The two components of the PCA explain 99.99% of the total variance (99.92% first, 0.07% second). As Figure 4 shows, the first principal component was highly correlated with TPC and negatively correlated with hydroxycinnamic acids and flavonols, and to a lesser degree with hydroxybenzoic acid. AA was in the second quarter, while the rest of the species studied were located in the third quarter of the biplot (except G in the middle line). This data representation shows that AA behaves differently from the rest of the kiwis studied, with higher content of TPC, hydroxycinnamic, and hydroxybenzoic acids compared to other commercial kiwi fruits.

Finally, Spearman correlation analysis was used to determine the correlation between the TPC, phenolic families, and antioxidant capacity. ABTS, ORAC, and Q-DPPH antioxidant capacity methodologies were positively correlated to each other (*p* = 0.05; r > 0.9) and showed a significant positive correlation with TPC (*p* = 0.05; r > 0.9). On the other hand, TPC and flavonols showed a significant and negative correlation (*p* = 0.05; r = −0.9). Moreover, flavonols were negatively correlated with ABTS, ORAC, and Q-DPPH (*p* = 0.05; r ≥ 0.9). These data suggest that the high TPC values of AA fruit may be responsible for its higher antioxidant capacity compared to other types of kiwi fruits.

## 3. Materials and Methods

### 3.1. Raw Material

We purchased approximately 600 g of samples from commercial markets in Madrid (Spain), corresponding to up to 40 fruits for each species/variety. *A. arguta* Miq. (AA) was from France, *A. deliciosa* (green) (AD) and *A chinensis* (gold) (G) were from New Zealand, and *A. deliciosa* cv. Hayward (green) (HW) and *A. deliciosa* cv. “Hayward eco” (ECO) (green; organic production) were from Spain. To facilitate handling and preservation, they were immediately freeze-dried (in darkness) using a Telstar S.A. lyophilizer (Spain), then milled, homogenized, and stored at −20 °C in a dark, dry environment to prevent the loss of labile compounds. After lyophilization, a portion of the samples was ground to obtain fine solid particles (0.037 mm size) to perform chemical analysis.

### 3.2. Chemicals

All chemicals were of analytical grade. AAPH (2,2‘-azo-bis (2-amidino-propano) dihidrocloruro) (ORAC), 1,1-diphenyl-2-picryl-hydrazl (DPPH), Folin–Ciocalteu reagent, 2,4,6,-tripyridyl-s-triazine (FRAP), Fast Blue BB [4-benzoylamino-2,5-dimethoxybenzenediazonium chloride hemi-(zinc chloride)] (total phenolic content, TPC), 2,21-azino-bis (3-ethylbenzothiazoline-6-sulphonic acid) diammonium salt (ABTS), phosphate-buffered saline (PBS), tert-butyl hydroperoxide (tBOOH), dimethyl sulfoxide (DMSO), 3-(4,5-dimethylthiazole-y)-2,5-diphenyltetrazolium bromide (MTT), and 21,71-dichlorodihydro-fluorescein diacetate (DCFH-DA) were purchased from Sigma Chemical (Sigma-Aldrich, St. Louis, MO, USA). Dulbecco’s Modified Eagle Medium (DMEM), L-glutamine, antibiotics (penicillin and streptomycin), and trypsin were from Gibco Laboratory (Invitrogen Co., Grand Island, NY, USA), and fetal bovine serum (FBS) was from Hyclone (GE Healthcare, Chicago, IL, USA).

### 3.3. Antioxidant Properties

#### 3.3.1. Overall Antioxidant Capacity—Extraction-Dependent Methods

Briefly, 100 mg of each of the lyophilized samples were weighed and carried to a final volume of 10 mL of water. Then, the mixture was stirred at room temperature 15 min for extraction and filtered with a 0.45 µm Millex filter and freeze-stored until use. The final extract concentration was 10 mg/mL, and this extract (E) was used for antioxidant capacity analysis.

- **2,2′-Azinobis-(3-Ethylbenzothiazoline 6-Sulfonic acid) (ABTS) Assay**

The trapping capacity of cationic free radicals was evaluated using the method of radical ABTS^•+^ bleaching, described by Re et al. (1999) and modified by Oki et al. (2006) for its use in a microplate [35,36]. This method implicates the reaction between ABTS and potassium persulfate producing the blue/green ABTS^•+^ chromophore. The addition of antioxidants to the pre-formed radical cation reduces ABTS, and coloration changes. A greater or lesser bleaching occurs depending on the antioxidant capacity, the concentration of the antioxidant, and the duration of the reaction. Decoloration is expressed as the percentage inhibition of the ABTS^•+^ radical cation compared to the reactivity of GAE as a standard. Aqueous solutions of GAE (8.82–61.7 μM) were used for calibration and PBS (phosphate-buffered saline) and ABTS solutions were used as negative and positive controls, respectively. Extracts of the sample (E) were diluted 1:2 for this analysis. Then, 300 μL of sample, negative and positive control, and standard were charged in the wells of a 96-well plate. Absorbance was measured at 734 nm using a UV–visible spectrophotometer (BioTek Instruments, Winooski, VT, USA). All measurements were performed in triplicate; results are expressed as mg of gallic equivalents (GE)/g sample.

- **Oxygen Radical Absorbance Capacity (ORAC) Assay**

The ORAC assay was used according to the method described by Ou et al. (2001) and as modified by Dávalos et al. (2005) [37,38]. It is based on the inhibition of reactive oxygen species (ROS) from the thermal decomposition of the 2,2’azobis amidinopropane dihydrochloride (AAPH) compound. During the assay, ROS from AAPH will decrease the fluorescent signal of fluorescein (FL) due to its oxidation. The addition of antioxidant compounds produces a more stable fluorescent signal, which is compared with a Trolox standard (5–85 μM). In the procedure, 20 μL PBS + 120 μL FL was used as a positive control, and 20 μL PBS + 120 μL FL + 60 μL AAPH was used as a negative control. The procedure was carried out using an automated plate reader (BMG LABTECH, Germany) equipped with a fluorescence detector set at excitation and emission wavelengths of 485 nm and 530 nm, respectively. Readings were taken every minute, for a total duration of 90 min, at 37 °C. Extracts of the sample (E) were diluted 1:10 for this analysis and 20 μL sample + 120 μL FL + 60 μL AAPH was charged in the wells. All measurements were performed in triplicate; results are expressed as μg of TE/g sample.

#### 3.3.2. Overall Antioxidant Capacity—Direct Methods: QUENCHER

Analyses were performed using the QUENCHER procedure (QUIck, Easy, New, CHEap, and Reproducible), applied to Folin–Ciocalteu, DPPH, and FRAP methodology, avoiding solvent extraction or a hydrolysis step, given that the reagents act directly on the sample.

- **Folin–Ciocalteu Assay by QUENCHER method**

The Q-FC method, which uses a reagent containing phosphomolyb-dic/phosphotungstic acid complexes, has been proposed as an antioxidant capacity analysis method rather than for the quantification of phenolic compounds [39,40], since not only phenolics, but also other reducing compounds such as ascorbic acid, may react with molybdenum, forming blue complexes that can be detected spectrophotometrically. Therefore, the methodology described by Slinkard and Singleton (1997) was adapted, weighing 1 mg of dried sample by triplicate [41]. Tubes were covered with aluminum foil, and 0.8 mL of distilled water and 0.2 mL of Folin–Ciocalteu phenol reagent were added and stirred in a vortex. After 5 min of reaction, 4 mL of Na_2_CO_3_ 0.7 M and 5 mL of distilled water were added. After stirring in vortex and 45 min of orbital agitation, absorbance was measured at 750 nm against a blank. The concentration of the calibration curve was 50–400 µg/mL. All measurements were performed in triplicate; results are expressed as mg of GAE/g sample.

- **1,1-diphenyl-2-picryl-hydrazl (DPPH) Assay by QUENCHER method**

The antioxidant capacity by the Q-DPPH method was determined following the methodology proposed by Del Pino-Garcia et al. (2015) [18]. Reaction mechanism is based on an electron transfer reaction and in this method, the purple chromogen radical 2,2-diphenyl-1-picrylhydrazyl (DPPH•) is reduced by antioxidant/reducing compounds to the corresponding pale yellow hydrazine. To a 0.1 mM DPPH solution, ethanol/water (50:50, *v*/*v*) was added to have an absorbance at 517 nm between 0.75 and 0.80. Then, using a precision scale (BOECO Germany), each sample was weighed (2 ± 0.1 mg) by triplicate in Falcon tubes. They were covered with aluminum foil and 10 mL of the DPPH dilution were added. Afterwards, they were stirred in a vortex (Velp Scientifica, Usmate, Italy) and kept under circular agitation for 1 h. After being centrifuged for 5 min at 7000 rpm and filtered by gravity, the absorbance at 517 nm was read in a Synergy HTX multi-mode reader spectrum using microplates. Trolox was used as standard to perform a calibration curve (12.5–200 µg/mL), and results were expressed as mg of Trolox equivalents (TE/g).

- **2,4,6-tripyridyl-s-triazine (FRAP) Assay by QUENCHER method**

The methodology described by Benzie and Strain (1996) was used for the determination of the Fe (III) reduction capacity by Q-FRAP analysis, with modifications from Del Pino-Garcia et al. (2015), at 595 nm [18,42]. Each sample was weighed (2 mg). Then, 40 mL of FRAP reagent was added and incubated at 37 °C for 30 min with continuous stirring. The absorbance was measured in a Synergy HTX multi-mode reader spectrum using microplates after being centrifuged for 5 min at 7000 rpm and filtered by gravity. Trolox was used as a standard to construct a calibration curve (concentration: 2.125–250 µg/mL), and the results were expressed as milligrams of TE per gram of product (mg TE/g).

#### 3.3.3. Intracellular Reactive Oxygen Species (ROS)

Normal rat small intestine epithelial cells (IEC-6) were cultured as a monolayer in Dulbecco’s Modified Eagle Medium (DMEM), supplemented with 10% (*v*/*v*) heat-inactivated fetal calf serum (FBS), 50 U/mL penicillin, 50 μg/mL streptomycin, and 1% (*v*/*v*) L-glutamine at 37 °C and in 5% CO_2_ in a humidified incubator (BINDER CB series 2010, Tuttlingen, Germany).

Prior to the study of basal intracellular ROS, the effect of different concentrations of kiwi extract on cell viability was measured by the MTT (3-[4,5-dimethylthiazol-2-yl]-2,5-diphenyl tetrazolium bromide) assay [43] in order to select non-cytotoxic doses. IEC-6 cells were treated with aqueous extract of kiwi samples at 0.25, 0.5, and 1 mg/mL. DMSO (50%) was used as a death control.

The determination of basal intracellular ROS was performed, following the same procedure used by Iriondo-DeHond et al. (2019) [44]. Briefly, sterile 96-well plates were seeded and incubated for 24 h with 20,000 cells/well. Then, samples at 1 mg/mL were added to each well. After 24 h of sample incubation, 2 µL of DCFH-DA probe (5 mg/mL in DMSO) was added to each well and incubated for 30 min. Then, samples at the same concentration were added and fluorescence was measured after 30 min of incubation in a fluorimeter microplate reader (λ_excitation_ = 485 nm, λ_emission_ = 528 nm). Non-treated control cells were considered to have 100% intracellular ROS. Tert-butyl hydroperoxide (tBOOH) at a concentration of 1 mM was used as a positive oxidation control, and ascorbic acid (10 μg/mL) was used as an antioxidant reference compound. Then, an MTT assay was performed to normalize the data by the number of cells per well. Experiments were carried out in triplicate.

Induced intracellular ROS were also measured when combining samples and tBOOH 1 mM. For the determination of induced intracellular ROS, cells were pre-treated with samples at 1 mg/mL for 24 h, and after the DCFH-DA probe incubation, t-BOOH 1 mM and samples (1 mg/mL) were co-administrated for 30 min. Then, fluorescence was measured and a final MTT assay was carried out.

### 3.4. Phenolic Compounds

#### 3.4.1. Total Polyphenols: Fast Blue (TPC) Assay by QUENCHER Method

The determination of total polyphenols (Q-Fast Blue BB) was carried out according to the methodology described by Arias-Rico et al. (2020) and Pedro et al. (2022) in order to avoid the interferences of reducing compounds with the FC method [45,46]. This method is based on the direct interaction between phenolics and the diazonium salt, present in the reagent Fast Blue BB, but ascorbic acid and other reducing compounds do not react with the diazonium salt, this method being the best choice for measuring TPC in foods containing reducing substances. A total of 2 mg of sample was weighed. Tubes were covered with aluminum foil and we added 0.4 mL of Fast Blue BB 0.1%, 0.4 mL of NaOH 5%, and 4 mL of distilled water, then stirred in vortex after all the additions, and left in orbital agitation for 45 min. Absorbance was measured at 420 nm on a microplate with a Synergy HTX multi-mode reader spectrum after being centrifuged for 10 min at 6500 rpm and filtered by gravity. Results were compared with a gallic acid calibration curve (concentration 5–200 µg/mL) and were expressed as an equivalent amount of this compound (GAE).

#### 3.4.2. Flavonols by QUENCHER Methodology

The determination of flavonols was adapted from Bonoli et al. (2004) [47]. Sample was weighed (10 mg) by triplicate, 0.5 mL of distilled water and 4 mL of methanol were added, and the sample was incubated at room temperature in an orbital shaker. After 15 min, the sample was centrifuged and filtered, and the absorbance was measured at 370 nm. Results were expressed as milligrams of quercetin equivalent per g (mg QE/g) using a calibration curve with different concentrations (0.5–25 mg/g) of the standard.

#### 3.4.3. Hydroxycinnamic Acids by QUENCHER Methodology

The determination of hydroxycinnamic acids was carried out according to Bonoli et al. (2004) with some modifications [47]. First, 0.5 mL of distilled water and 4 mL of methanol were added to the sample (10 mg); then, it was homogenized and incubated for 15 min in an orbital shaker. After this, the sample was centrifuged and filtered, and finally, the absorbance was measured at 320 nm. All determinations were made in triplicate. A ferulic acid calibration (0.25–25 mg/g) curve was obtained, and the results were expressed as milligrams of ferulic acid equivalent per gram (mg FAE/g).

#### 3.4.4. Hydroxybenzoic Acids by QUENCHER Methodology

The hydroxybenzoic acid determination was adapted from the method described by Bonoli et al. (2004) [47]. First, 0.5 mL of distilled water and 4 ml of 3% formic acid were added to the sample (10 mg) and homogenized with a vortex. After 15 min of incubation in an orbital shaker, the sample was centrifuged and filtered, and the absorbance was measured at 280 nm in quartz cuvettes. Each sample was analyzed in triplicate. The results were expressed as milligrams of gallic acid equivalent per gram (mg GAE/g) by means of a calibration curve with different concentrations of the standard.

### 3.5. Statistical Analysis

Triplicate analysis was performed for all the samples. In tables, the mean ± standard deviation (SD) of three replicates is given for all the measured parameters. Data were statistically analyzed by analysis of variance (ANOVA) followed by Duncan’s test. The statistical significance level was set at *p* < 0.05. Moreover, multivariate principal component analysis (PCA) was applied. PCA functions as an exploratory data analysis by projecting each data point onto only the first few principal components to obtain lower-dimensional data while preserving as much of the data’s variation as possible. Spearman multivariate correlation was also used (Statgraphics Plus 5.1 software, Warrenton, VA, USA).

## 4. Conclusions

The five types of commercial kiwi fruits analyzed in this study showed interesting values in terms of antioxidant capacity, with AA fruits showing the highest values in all the assays used, followed by AD for the ORAC and ABTS assays, as well as G for the Q-FRAP and Q-DPPH assays. The direct Q-FRAP method was applied to kiwi fruits for the first time in this study and best represented the antioxidant capacity of the samples, allowing the measurement of soluble and non-soluble antioxidants. AA fruits presented the highest values of TPC compared to the other kiwi fruits, as measured by the Q-Fast Blue method, with relevant amounts of hydroxybenzoic acids. According to PCA and correlation studies, the high TPC values that characterized AA fruits are significantly correlated to its higher antioxidant capacity.

This study also demonstrates that all the analyzed kiwi fruits inhibited induced ROS production in IEG-6 healthy intestinal cell lines, but dwarf kiwi fruits were the most efficient in the inhibition of physiological ROS production. Since fruits are essential foods in a healthy diet, dwarf kiwi is an exotic fruit that can highly contribute to the quality of current diets. In addition to being rich in phenolic compounds, these fruits present higher antioxidant capacity compared to conventional kiwi fruits, and they do not generate any waste, as the skin and peel can be consumed, making them a sustainable and healthy alternative to diversify fruit intake in the diet.

## Figures and Tables

**Table 1:** Overall antioxidant capacity of kiwi samples measured using different methodologies (extraction-dependent and direct by QUENCHER).

	Q-Folin–Ciocalteu (mg GAE/g)	Q-DPPH (mg TE/g)	Q-FRAP (mg TE/g)	ORAC (µg TE/g)	ABTS (mg GAE/g)
**AD**	4.09 ± 0.20 ^a^	6.41 ± 0.11 ^a^	9.28 ± 0.45 ^b^	3.94 ± 0.00 ^d^	1.45 ± 0.05 ^c^
**HW**	6.95 ± 0.35 ^b^	6.16 ± 0.20 ^a^	9.88 ± 0.20 ^b^	2.38 ± 0.00 ^a^	0.67 ± 0.02 ^a^
**ECO**	4.23 ± 0.22 ^a^	6.21 ± 0.35 ^a^	7.24 ± 0.31 ^a^	3.08 ± 0.00 ^b^	0.99 ± 0.02 ^b^
**G**	6.74 ± 0.56 ^b^	6.98 ± 0.62 ^b^	16.00 ± 0.78 ^c^	3.38 ± 0.00 ^c^	1.43 ± 0.11 ^c^
**AA**	10.82 ± 0.36 ^c^	16.31 ± 1.10 ^c^	26.24 ± 1.22 ^d^	4.75 ± 0.00 ^e^	1.73 ± 0.05 ^d^

Data are expressed as mean ± standard deviation (*n* = 3). In each column, different letters mean statistically significant differences (*p* < 0.05) compared by Duncan’s test. GAE: gallic acid equivalent; TE: Trolox equivalent; AD: *A. deliciosa*; HW: *A. deliciosa* var. Hayward; ECO: *A. deliciosa* var Hayard organic production; G: *A. chinensis* (gold fruits); AA: *A. arguta*. Data are expressed as dry weight.

**Table 2:** TPC (Q-Fast Blue) and phenolic families of kiwi varieties, expressed as mg/g (dry weight).

	TPC (mg GAE/g)	Flavonols (mg QE/g)	Hydroxycinnamic Acid (mg FAE/g)	Hydroxybenzoic Acid (mg GAE/g)
**AD**	32.13 ± 2.10 ^b^	1.26 ± 0.03 ^c^	0.32 ± 0.01 ^a^	2.42 ± 0.21 ^a^
**HW**	23.02 ± 2.38 ^a^	1.44 ± 0.10 ^d^	0.34 ± 0.03 ^a^	2.34 ± 0.07 ^a^
**ECO**	29.34 ± 2.35 ^b^	1.28 ± 0.07 ^c^	0.60 ± 0.02 ^c^	2.41 ± 0.07 ^a^
**G**	31.30 ± 2.61 ^b^	0.85 ± 0.01 ^b^	0.49 ± 0.01 ^b^	2.28 ± 0.13 ^a^
**AA**	54.57 ± 4.60 ^c^	0.55 ± 0.02 ^a^	2.39 ± 0.02 ^d^	2.40 ± 0.19 ^a^

Data are expressed as mean ± standard deviation (*n* = 3). In each column, different letters mean statistically significant differences (*p* < 0.05) compared by Duncan’s test. TPC: total phenolic content; GAE: gallic acid equivalent; QE: quercetin equivalent; FAE: ferulic acid equivalent. AD: *A. deliciosa*; HW: *A. deliciosa* var. Hayward; ECO: *A. deliciosa* var Hayard eco; G: *A. chinensis*; AA: *A. arguta*. Data are expressed as dry weight.

## Data Availability

Not applicable.

## Sample Availability

The samples analyzed are available from the authors.
